# Expandable Graphite as a Multifunctional Flame-Retarding Additive for Highly Filled Thermal Conductive Polymer Formulations

**DOI:** 10.3390/polym14081613

**Published:** 2022-04-15

**Authors:** Florian Tomiak, Kevin Schneider, Angelina Schoeffel, Klaus Rathberger, Dietmar Drummer

**Affiliations:** 1 Institute of Polymer Technology, Friedrich-Alexander-University Erlangen-Nuremberg, Am Weichselgarten 10, 91058 Erlangen, Germany; kevin.schneider@fau.de (K.S.); dietmar.drummer@fau.de (D.D.); 2 Bavarian Polymer Institute, Friedrich-Alexander-University Erlangen-Nuremberg, Dr. Mack Strasse 77, 90762 Fuerth, Germany; 3 Georg H. Luh GmbH, Schoene Aussicht 39, 65396 Walluf, Germany; angelina.schoeffel@luh.de (A.S.); klaus.rathberger@luh.de (K.R.)

**Keywords:** expandable graphite, flame retardant, thermal conductivity, highly filled polymers, thermal imaging

## Abstract

Expandable graphite (EG) and graphite (G) were assessed as multifunctional additives improving both flame retardancy and thermal conductivity in highly filled, thermal conductive polymeric materials based on polyamide 6 (PA6). Fire testing was conducted using modern UL-94, LOI and cone calorimeter test setups. It is demonstrated that thermal conductivity can significantly influence the time to ignition, although offering little fire resistance once ignited even in highly filled systems. Thus, for PA6 formulations containing solely 70 wt.% G, the peak heat release rate (pHRR) measured in cone calorimeter tests was 193 kW/m², whereas PA6 formulations containing 20 wt.% EG/50 wt.% G did not exhibit a measurable heat development. Particular attention was paid to effect separation between thermal conductivity and residue formation. Good thermal conductivity properties are proven to be particularly effective in test scenarios where the heat impact is comparatively low and the testing environment provides good heat dissipation and convective cooling possibilities. For candle-like ignition scenarios (e.g., LOI), filling levels of >50 wt.% (G or EG/G) are shown to be sufficient to suppress ignition exclusively by thermal conductivity. V0 classifications in UL-94 vertical burning tests were achieved for PA6 formulations containing ≥70 wt.% G, ≥25 wt.% EG and ≥20 wt.% EG/25 wt.% G.

## 1. Introduction

Polymers offer a wide variety of material properties and processing advantages compared to other material classes. They provide a low weight, high mechanical strength and good chemical resistivity, and can easily be processed economically into geometrically complex products. Electronic components or machine housings, for example, require not only complex component geometries, but also the dissipation of heat generated during use, as otherwise heat buildup can cause functional failures [1,2,3,4]. To improve their naturally low thermal conductivity characteristics (0.2–0.5 W/mK), polymers can therefore be modified by the incorporation of highly conductive particles such as copper [3,5], aluminum or graphite [6,7,8] with natural conductivity between 100 W/mK and 600 W/mK. At high incorporated levels of graphite fillers, polymeric materials have been reported to achieve a thermal conductivity of up to 30 W/mK [4,6], while retaining good molding characteristics.

Proximity to heat sources increases the risk of emerging fires. Many frequently used polymers are naturally highly flammable and combustion may contribute significantly to heat generation and can be responsible for strong smoke development [9]. The most common approach to reducing the risk of potential fire hazards and meeting stringent protection standards is the incorporation of flame-retardant additives into a polymeric material. Depending on the type of active ingredient, flame-retardant additives act by dilution, cooling, residue formation or flame poisoning [9,10,11,12]. In order to achieve high flame retardancy classifications, considerable weight fractions of flame retardants are often required, reducing the potential space available for other functional additives or fillers. Expandable graphite offers a rare combination of multifunctional characteristics combines both flame-retardant and thermal conductivity properties. Like graphite, expandable graphite consists of stacked graphene platelets bound by Van-der-Waals forces, whose nanometer-thin spaces may be intercalated by a blowing agent [13]. When exposed to heat, the layers are forced apart, resulting in characteristic worm-like structures [14,15,16]. The thermal degradation of polymeric materials containing expandable graphite leads to the formation of a voluminous, thermally stable char layer at the surface of the polymer. This char inhibits heat feedback from the combustion zone and reduces the rate of formation of volatile fuel fragments to feed the combustion process. The efficiency of the flame-retardant effect is largely dependent on the expansion volume provided by the incorporated graphite particles, which have been reported to be mainly controlled by their initial size [17]. Since expandable graphite acts solely physically, fire inhibitation effects are similar for various polymeric systems. The main commercially available products modified by EG are flame-retardant coatings and polyurethane foams, but EG has been reported to be efficient in various polymeric material systems (e.g., PE [18,19,20,21], PP [22,23], PS [24], PVC [25], ABS [26], PA6 [27,28,29,30]).

A comprehensive analysis of the burning characteristics of thermally conductive formulations based on PA6, graphite (G) and expandable graphite (EG) was carried out. Insights regarding the impact of thermal conductivity on flame-retardant properties and classifications reflected in cone calorimeter, limiting oxygen index (LOI) and UL-94 (Underwriter Laboratory certified fire test) fire tests were obtained. Furthermore, thermal conductivity influences were evaluated independent of isolation effects provided by a char layer at the surface of the degrading polymer provided by EG. Microscopy imaging and thermal conductivity measurements were conducted to analyze particle orientation and its impact on basic thermal conductivity. Thermal analysis was used to obtain insights into decomposition temperatures and characteristics. Additional infrared imaging was used to evaluate flame impact and cooling properties for selected samples in realistic testing scenarios. The results presented might be particularly useful for applications in which thermally conductive species may be used as part of a flame-retardant formulation.

## 2. Materials and Methods

### 2.1. Materials and Preparation

A PA6 grade B27E from BASF SE (Ludwigshafen, Germany), an expandable graphite GHL PX 95 HT 270 and a graphite type GHL 3394 from LUH GmbH (Walluf, Germany) were used within this study (Table 1). All polymeric materials were produced by a twin-screw compounder (co-rotating), the DSE ZSE HP 27 by Leistritz GmbH (Nuremberg, Germany), using two gravimetrical feeder units for G and EG. Polymeric materials containing filling degrees lower or equal to 50 wt.% were drawn off via water bath, granulated and dried afterwards. For polymeric material containing higher weight fractions, strands were too brittle. Thus, a cooled chute was used and the strands chipped afterwards. No drying was necessary for these polymeric materials. Samples were prepared by injection molding using an injection molding machine, the Arburg Allrounder 370 V by Arburg GmbH & CoKG (Loßburg, Germany). The cylinder temperatures were controlled between 230 °C (die) and 220 °C, the injection speed was 60 mm/s and the mold temperature 80 °C. Subsequent to injection molding, samples were prepared to fit the following sample geometries: Cone calorimeter samples 100 × 100 × 4 mm; LOI samples 125 × 10 × 1, 2, 4 mm³; UL-94 125 × 13 × 1, 2, 4 mm³ and samples for infrared imaging tests 125 × 13 × 1, 2, 4 mm³. All samples were dry-conditioned (70 °C vacuum) until weight consistency was reached.

### 2.2. Microscopy

Reflected light microscopy images were taken using an AxioImagerM2m by Zeiss AG (Oberkochen, Germany). Samples were prepared by embedding in a transparent epoxy resin, subsequent grounding and polishing. Microscopy images were taken to identify filler distribution, orientation and potential processing defects.

### 2.3. Thermal Analysis

A STA F3 449 Jupiter by Netzsch (Selb, Germany) was used for the combined study of thermal gravimetric analysis (TGA) and differential scanning calorimetry (DSC). TGA was conducted under non-isothermal conditions. All samples were heated between 50 °C and 800 °C at a N2 flow rate of 70 mL/min at 20 K/min. Due to high end temperatures, the sample carrier (TG-DSC) was equipped with aluminum oxide tilts. Sample weights were constant at 10 ± 1 mg. The onset temperature is defined as 99% residual mass. All tests were conducted at least two times. Averaged curves are presented.

### 2.4. Thermal Conductivity, Density and Heat Capacity Measurements

Heat conductivity measurements were performed using a Nanoflash device, LFA 447 by Netzsch GmbH (Selb, Germany) at room temperature. Sample geometries were 12.7 × 12.7 × 4 mm^3^. In order to measure thermal conductivity in plane direction (x), stripes were prepared from injection-molded plates, turned perpendicular, glued and milled to fit geometrical standard requirements of 12.7 × 12.7 × 4 mm^3^.

For density measurements, a gaspyknometer device, the AccuPyc 1330 by Micromertics Instrument Corp. (Norcross, GA, USA) was used. For heat capacity measurements, a Calvet Calorimeter C80 by Setaram Instrumentation Inc. (Caluire, France) was used. All samples were dried before testing and measured in accordance to standards.

### 2.5. Fire Testing

Fire behavior was analyzed using a Cone Calorimeter (ISO 5660-1), UL-94 (DIN EN 60695-11-10/20) and Limiting Oxygen Index (LOI) (DIN EN ISO 4589-2); all devices were manufactured by Netzsch Taurus GmbH (Weimar, Germany). Cone Calorimeter tests are used to monitor the heat generation and smoke density of materials in fully developed fire situations. Results are represented as the time-dissolved function of (1) the heat release rate (HRR) traced by the O_2_ consumption, (2) a signal value of the pyrolysis gas transparency smoke production rate (SPR) traced by a laser and the mass loss rate (MLR) traced by a scale. The heat release characteristics are unique for different material systems and can thus be used to identify flame inhibitation effects. Since a comparison of entire curves is not practicable, average values, maximum values, sum values as well as ratio values are frequently used as comparative parameters. The most important key figures are: HRR, peak of the heat release rate (pHRR), total heat emitted (THE), MLR, total mass loss (TMLR), time to ignition (T_ign_), total smoke production (TSP), average rate of heat emission (AHRE) or the maximum AHRE (MAHRE) [27,28,29]. Samples used within this study were 100 × 100 × 4 mm^3^ and tested at external heat fluxes of 50 kW/m^2^. All tests were repeated at least three times. Averaged curves are presented. For a sample selection, three thermocouples were placed diagonally on the lower specimen side in order to evaluate the thermal isolation effect given by char residue formation as well as thermal conductivity effects distributing heat throughout the material system. Care was taken to locate the measuring positions outside the metal brim [31,32,33].

Limiting Oxygen Index (LOI) measurements are used to assess the flammability properties of polymeric materials. The test setup provides a candle-like ignition scenario. Within the testing routine, a 50-watt propane flame is applied six times for five seconds to the upper end of a vertically clamped sample. During the measurement, the atmospheric oxygen content is adjusted in an event-controlled rhythm. The resulting oxygen content is converted to an oxygen index (OI) and represents the minimum oxygen content required to maintain a flaming combustion [34].

UL-94 burning tests measure self-distinguishing properties of polymeric materials. Compared to LOI measurements, UL-94 testing scenarios are more severe, since a 50-watt flame is applied underneath a vertically clamped sample. The sample is exposed twice for 10 s and specific burning characteristics are observed. Three classifications, V0, V1, V2 are then to be assigned based on observations conducted from the tests. The highest classification is represented by V0, which corresponds to a non-burn-dripping and almost instant self-extinguishing behavior. V1 classifications allow longer afterburning, but prohibit burn-dripping. The lowest classification, V2, is mostly considered insufficient, since self-extinguishing behavior is only moderate and burn-dripping occurs. All tests were conducted in accordance to standards [35].

### 2.6. Infrared Imaging

Heating processes typical for horizontal UL-94 test setups given by a 50 W flame exposure and cooling processes after flame removal were investigated by infrared imaging. An infrared camera with an integrated CO_2_ filter, VarioCAM 880 HD Head by InfraTec GmbH (Dresden, Germany), was used, allowing the measurement of surface temperatures by excluding the visual flaming zone infrared spectra. The emission coefficient was calibrated to 0.93. Two test scenarios were defined to measure thermal dissipation effects within realistic testing scenarios: (1) LOI-like test setup: A 50 W propane testing flame was applied in a candle-like setup for 120 s under atmospheric conditions. Temperature measurements were conducted during flame exposure as well as after flame removal in order to observe both heating and cooling processes. Sample geometries were 125 × 13 × 4 mm³. All tests were repeated three times. (2) UL-94 test setup: A 50 W methane testing flame was applied underneath a vertically mounted specimen for 2 × 15 s under atmospheric conditions. Temperature measurements were conducted during flame exposure as well as after flame removal in order to observe both heating and cooling processes. Sample geometries were 125 × 13 × 4 mm³. All tests were repeated three times.

## 3. Results

### 3.1. Microscopy

**Figure 1:**
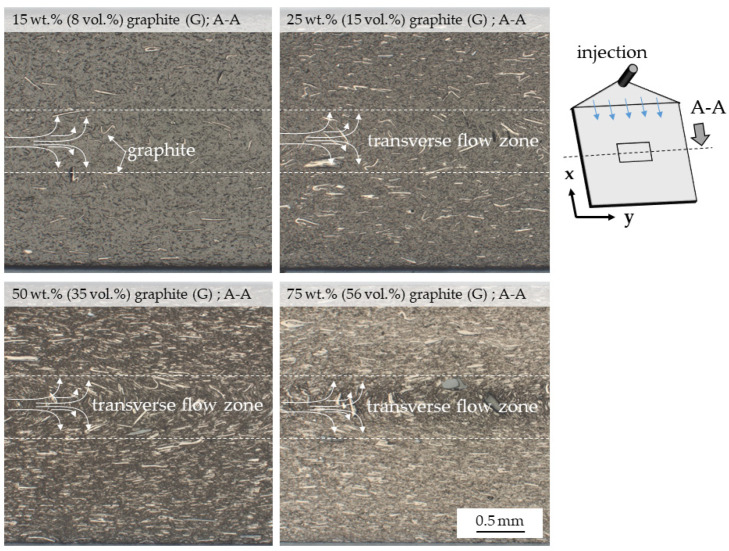
Microscopy images of graphite-filled PA6. Images were taken perpendicular to the injection-flow, as illustrated.

Microscopy images show a homogeneous graphite particle distribution, with no visible voids or cracks (Figure 1). The particle orientations observed can be visually divided into three layers, whereby the distinction is particularly clear at higher filling degrees. This three-layer characteristic (a, b, a) is well known for thermoplastic polymers and has been widely discussed in the literature [5,36].

Particles located in the boundary layers are oriented predominantly in molding direction, while those in the core layer occur transversely. Unlike thermoset molding polymeric materials, thermoplastics form a fountain-like flow characteristic while molding, which automatically aligns floating particles and fibers. Proportions between core and boundary layers have been reported to be predominantly dependent on the viscosity properties given by the polymeric material system. For the given formulations, the core- layer-to-total-thickness ratio was found to be around 80%, which fits well with previously reported values [36].

### 3.2. Thermal Analysis—TGA and DSC

**Figure 2:**
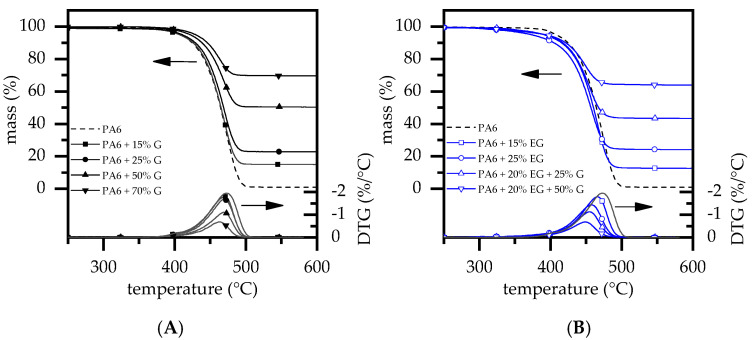
TGA analysis results for (**A**) PA6/G and (**B**) PA6/EG and PA6/G/EG formulations. Measurements were conducted under nitrogen atmosphere; a heating rate of 20 K/min was applied.

TGA/DSC measurements exhibited a single decomposition step for all formulations tested (Figure 2). PA6 is known to vaporize mainly in one decomposition step when tested under inert conditions. Measurements revealed a decomposition onset at 385 °C, a DTG peak at 472 °C and 0.9% residue. This fits well with values previously reported [28,29,37,38,39,40]. When G was added, residues measured increased directly proportionally to the filling degree present, with no observable change in general gravimetrical decomposition characteristics (Figure 2A).

Thus, the decomposition onset and DTG peaks were found in a range of 385–392 °C (T_99%_) and 464–472 °C (DTG-peak), respectively. For PA6 formulations containing EG as a flame-retardant additive, the decomposition onset temperatures decreased due to an earlier reaction mode. Since the DTG-peak remained at similar temperatures and no further decomposition steps occurred, no major changes within the reaction profile were to be expected. This fits well with a recently published study [28]. Values measured within this study can be found in Table 2.

### 3.3. Thermal Conductivity Analysis

**Figure 3:**
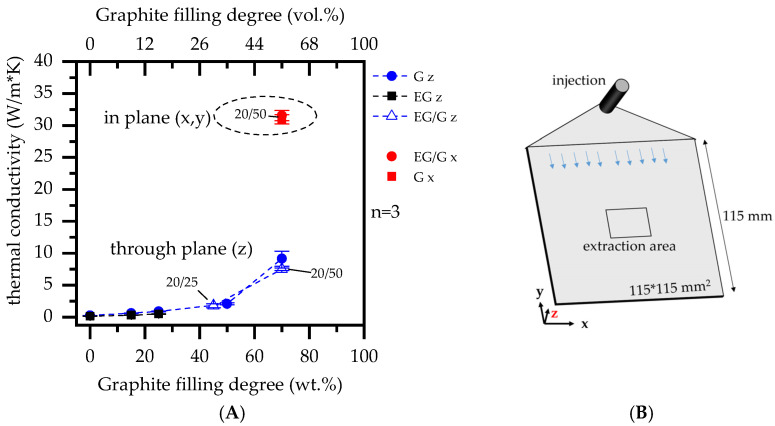
(**A**) Thermal conductivity measurements (4 mm) in (x) and thru-plane (z); (**B**) Illustrated injection molding plate and specimen extraction area.

As expected, larger incorporated fractions of graphite showed an increasing improvement of thermal conductivity properties (Figure 3). No substantial differences were found between the graphite grades EG and G. As reported in the literature, graphite exhibits anisotropic properties due to its platelet shape, providing superior thermal conductivity in plane direction. Accordingly, the highest values were measured for PA6 formulations containing 70 wt.% G and 20 wt.% EG/50 wt.% G with 7.5 ± 0.2 W/mK/9.2 ± 0.5 W/mK through-plane (z) and 31.0 ± 1.1 W/mK and 31.5 ± 1.0 W/mK in plane (x), which are in good agreement with values that have been reported in the literature [6,7,8]. Results are additionally listed in Table 3.

### 3.4. Fire Testing—Cone Calorimeter

**Figure 4:**
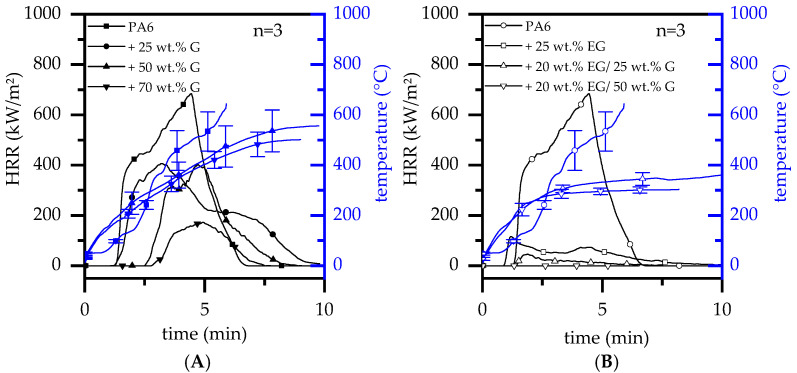
Cone Calorimeter results for a recipe selection; EG: expandable graphite, G: Graphite; (**A**) PA6/G formulations (**B**) PA6/EG and PA6/EG/G formulations.

When G was gradually incorporate into a PA6 matrix, burning characteristics as observed in cone calorimeter tests changed significantly. The flame retardant mode of action given by graphite is predominantly passive by polymer substitution. However, higher filling degrees tend to form a thermally stable non-voluminous char residue, somewhat limiting the external heat impact and combustion fueling process. This is particularly evident in heat development over time, reducing the pHRR and THE (Figure 4A). For filling degrees ≥50 wt.% G, an ignition time (t_ign_) delay could be observed. This effect can be attributed to a denser residue formation, allowing combustion gases to escape through local cracks. Since these do not necessarily arise below the piloted ignition source, combustion gases are partially diverted, so that ignition occurred randomly over time. As a consequence, higher standard deviations were obtained for the measured ignition time (t_ign_). Important key figures are listed in Table 4.

When a critical heat flux was applied, incorporated EG expanded to multiples of its original size, forming a volumetric, thermally stable char residue on the material surface. The residue acts as thermal barrier, shielding lower polymer layers from direct heat exposure, which in turn limited fuel generation over time. Thus, PA6 containing gradually increasing filling degrees of EG exhibited a decreasing trend in heat development, particularly evident in a reduction of the pHRR and THE (Table 4). These findings fit well with values previously reported in [28]. Similar effects were found for formulations containing 20/25 wt.% EG/G and 20/50 wt.% EG/G. The incorporation of G further substituted polymeric fractions. As a consequence, heat development further decreased, exhibiting a very low pHRR for 20/25 wt.% EG/G formulations (50 ± 14 kW/m²) and no detectable flaming combustion for 20/50 wt.% EG/G formulations (Table 4). Thus, expansion combined with fuel substitution and barrier effects, as previously outlined for PA6/G formulations, exhibited a superior flame retardancy behavior.

Residue formation as well as its thermal barrier effect is evident by tracking heat development at the lower side of the specimen. Three thermocouples were therefore diagonally attached to the lower specimen side and used for temperature tracking during standard cone calorimeter tests. Recorded temperature curves were averaged and are also presented in Figure 4.

Since a thermally insulating residue only forms over a longer period of external heat exposure, the temperature development observed was initially similar for all formulations tested. During the build-up process, a more voluminous, thermally isolating residue was formed, which subsequently changed temperature profiles measured directly proportionally to isolating efficiency. In other words: the more thermal isolation capabilities are gained by char residue formation, the slower the rise in temperatures will be at the lower specimen side. Despite a stronger residue formation being observed for PA6/G formulations containing high filling degrees (50 wt.% and 70 wt.%), it is evident that these do not provide good long-term thermal isolation. Temperatures measured increased and eventually exceeded temperatures well over the polymer decomposition range (>400 °C). A different temperature development was observed for EG containing formulations. From an initially similar heat development, temperatures developed regressively once a critical residue had been built. Thus, temperatures did not exceed 400 °C, indicating superior long-term isolation capabilities.

**Figure 5:**
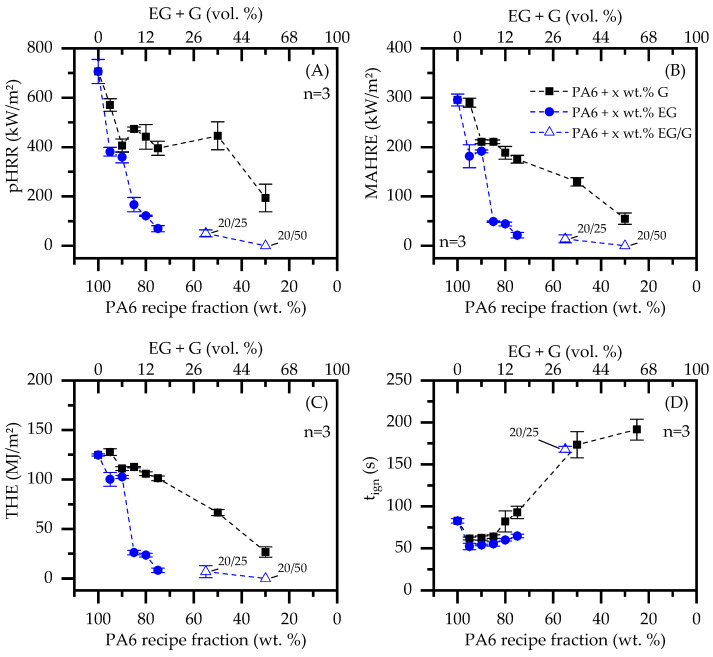
Important Cone Calorimeter key figures plotted against the PA6 weight fraction; (**A**) peak heat release rate (pHRR), (**B**) maximum of the average rate of heat release (MAHRE), (**C**) total heat emitted (THE) and (**D**) ignition time (t_ign_).

When cross-referencing key figures conducted by cone calorimeter tests with the remaining polymer fractions, active flame inhibitation effects can be separated from passive effects caused by polymer substitution (Figure 5). Formulations based solely on G show an indirect proportional behavior relative to filler content. Thus, the pHRR, MAHRE and THE dropped almost linearly with a decreasing polymer fraction. This proves an almost exclusive key-figure dependence on the polymer fraction present and thus limits flame inhibitation effects solely to fuel substitution. A quite different behavior is shown by the temporal shift of the ignition temperature (t_ign_). As discussed earlier, this effect is gained artificially by an increasingly stable, non-voluminous residue, only locally releasing combustion gasses when cracks occur. Even though combustion gases postpone piloted ignition by bypassing, the given residue effectively hinders an accelerated fire development once ignited.

PA6/EG and PA6/EG/G formulations on the other hand show a disproportionate reduction in key figures. Since thermally isolating residues decrease the heat impact and thus decelerate combustion processes, cone calorimeter tests are particularly sensitive to residue formation. Results showed a significant reduction in all key figures when the EG fraction was increased. pHRR, MAHRE and THE decrease disproportionately with the reduction of the polymer weight fraction, reaching particularly low values for filling degrees equal to or greater than 15 wt.% EG. When EG was added to 25 wt.% and 50 wt.% G, the flame-inhibiting effect was mostly provided by residue formation. As for PA6/G formulations, further key figure improvements between 20 wt.% EG and 20 wt.% EG/50 wt.% G seemed to be dependent solely on polymer substitution.

**Figure 6:**
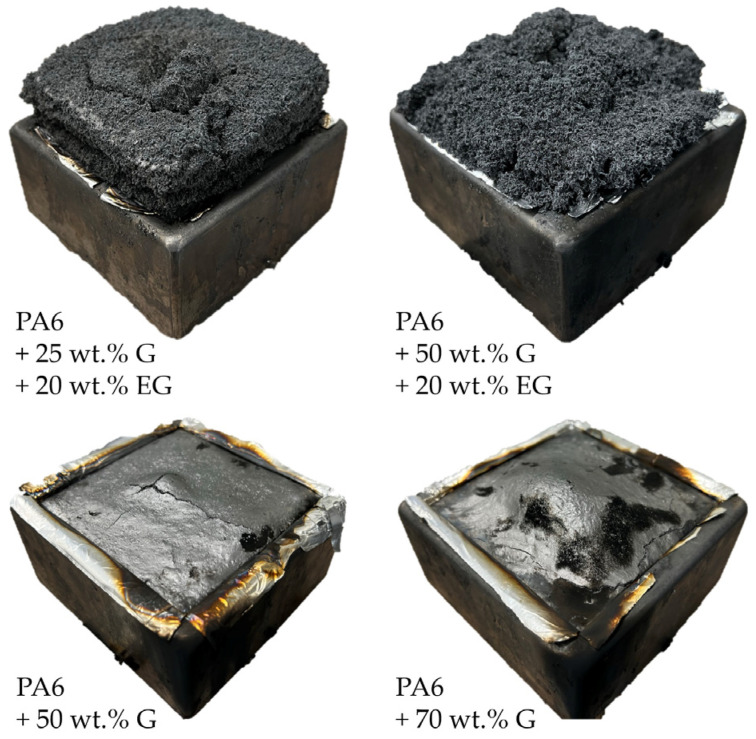
Selected images of char residues after cone calorimeter testing.

The effect of expandable graphite as a flame-retardant additive in PA6/EG/G formulations becomes particularly clear when comparing residues evident after cone calorimeter testing (Figure 6). PA6/G formulations form a dense, non-voluminous residue, which can be characterized by a glossy surface only disturbed by some local cracks. Variations in filling degrees did not cause substantial changes in the visual appearance. PA6 formulations containing EG, on the other hand, showed a voluminous residue formation exhibiting a porous system of expanded graphite particles. In contrast to the dense residue presented by PA6/G formulations, pyrolysis gases can evaporate easily, explaining the earlier ignition time observed. It is obvious that residue expansion comes at the expense of residual density. Therefore, it is not surprising that in order to gain a good flame inhibiting effect, a minimum degree of thermal insulation must be provided by a sufficient residue volume. When comparing PA6 containing 25 wt.%/20 wt.% G/EG versus 50 wt.%/20 wt.% G/EG, some differences in residue characteristics were also evident. Despite identical EG fractions, higher filling degrees resulted in a more irregular residue with a less voluminous characteristic. We attributed this to two reasons: (1) In more highly filled systems, particles have less individual space available, limiting spatial freedom for expansion. The expansion process is thus irregularly hindered and might even be prevented in some cases. (2) Another explanation might be an induced sheer stress through particle–particle interactions, resulting in particle size reduction, which subsequently reduces potential expansion properties. This hypothesis must be investigated in further studies and cannot be evaluated conclusively within this paper.

Cone calorimeter testing environments provide a well-controlled testing environment, whereas samples are almost completely isolated. Significant heat dissipation, which might influence burning characteristics through convective and conductive processes, does not occur. Consequently, it is concluded that heat conductivity is not relevant in fire scenarios prohibiting heat dissipation. However, other test scenarios allow heat dissipation and thus might affect flammability properties through cooling effects. This will be discussed in the following sections.

### 3.5. Fire Testing—LOI and UL-94

LOI and UL-94 fire tests are designed to measure the flammability and self-extinguishing capabilities of polymeric materials. Since only a small part of a specimen is exposed to a comparatively small flame, accumulated heat can be transferred within the specimen geometry from the impact zone towards lower temperature areas. Lower temperatures of the surrounding atmosphere enable subsequent cooling through conductive heat exchange, following a heat source/heat sink model.

**Figure 7:**
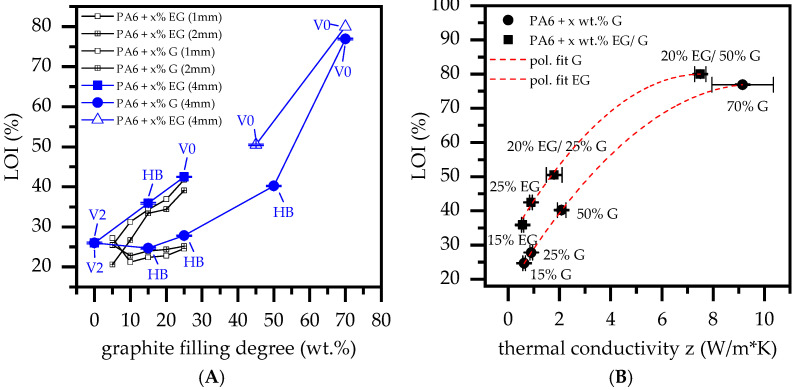
LOI and UL-94 testing results; (**A**) sample thickness 1 + 2 + 4 mm, (**B**) correlation study of thermal conductivity versus LOI.

PA6 characteristically possesses a low melt viscosity, which in the event of fire exposure allows a melt flow to leave the burning area. Since UL-94 and LOI tests are sensitive to this characteristic, net PA6 achieved a V2 classification as well as a comparatively high oxygen index of 26% for 4 mm thick samples (Figure 7A). Lower sample thicknesses did not achieve any UL-94 classification as the flame spread too quickly. The addition of fillers, such as graphite, reduce the melt flow ability and thus prevent self-extinguishing properties, as evident for net PA6. For filling degrees between 5 wt.% and 25 wt.% G in a PA6/G formulation, the oxygen index increased linearly from around 21% to 25%. Comparing sample geometries, thicker samples showed slightly higher oxygen indices.

Besides 2 mm thick samples containing 10 wt.% G, which achieved a V2 classification caused by melt breaking, no extinguishing behavior could be identified. For higher filling degrees of 50 wt.% and 70 wt.% (thickness: 4 mm), the oxygen index increased significantly to 40% and >70%, although only the latter achieved a V0 classification. Please note:

- Despite no UL-94 classification being achieved for PA6 containing 50 wt.% graphite, ignition was clearly suppressed. Not all samples out of the five-sample-set burned, although once ignited, no extinction occurred.
- PA6 containing 70 wt.% did not ignite under testing conditions. However, if exposure times exceeded 2 × 10 s, ignition occurred eventually. No self-extinguishing properties could be observed after ignition.

**Figure 8:**
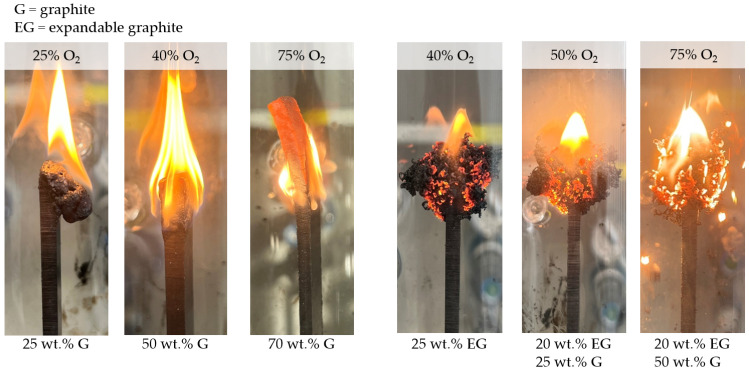
LOI images—characterization and comparison of burning behavior in dependence of the filling degree.

PA6/EG formulations generally exhibited superior LOI values. For filling degrees of 5 wt.% to 25 wt.%, the LOI measured increased from 20% to 43%, achieving V0 classifications for 25 wt.% EG. Samples with 1 mm thickness seemed to achieve slightly superior LOI values, whereas no difference in UL-94 classifications could be identified. Since expansion volume exceeded the optimum between 20 wt.% and 25 wt.%, EG/G were combined for higher filling degrees, whereas the EG weight fraction was set to 20 wt.%. Both PA6 containing 20 wt.% EG/25 wt.% G and 20 wt.% EG/50 wt.% G achieved V0 classification, with no ignition occurring even when exposure times exceeded 2 × 10 s many times over. LOI values measured were 50% and >70%, although this was difficult to measure since graphite particles started to oxidize at high oxygen levels (Figure 8). A summary of all LOI values and UL-94 classifications achieved can be found in Table 5.

When correlating LOI values against thermal conductivity properties (thru-plane), a good correlation was found for filling degrees between 15 wt.% and 50 wt.% G (Figure 7B). Formulations containing 70 wt.% did not correlate linearly, but resulted in a rather regressive characteristic. This is little surprising, since the testing atmosphere at this point mostly consisted of oxygen, and thus, graphite started to oxidize (Figure 8). A similar development could be identified for EG containing formulations. Since the heat input into LOI tests is initially low, EG particles expand with a certain time delay.

Once ignition has occurred, residue formation proceeds rapidly, causing the specimen to extinguish. These self-extinguishing properties ensure that EG-containing formulations exhibit superior characteristics to PA6/G formulations in LOI test setups, consequently reaching higher LOI values. When comparing LOI values relative to thermal conductivity properties, EG-containing formulations showed similar correlations as found for PA6/G formulations, yet on a higher general level. The corresponding gap between LOI values measured for PA6/EG and PA6/G recipes refers to the thermal barrier given by a strong expansion of incorporated EG. Interestingly, the measured oxygen index delta is more or less constant at ~10%.

### 3.6. Thermal Conductivity in Fire Testing Environments—Thermal Imaging

In order to evaluate thermal conductivity effects in real testing environments, thermal imaging was used in UL-94- and LOI-like testing scenarios. Ignition tests were recorded by thermal imaging and analyzed afterwards, using maximum temperatures for comparative reasons. Detailed information on the testing setup and procedure can be found in Chapter 2.5.

**Figure 9:**
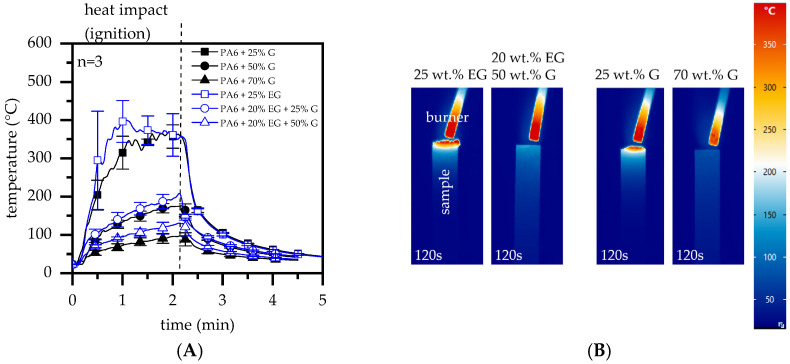
Thermal imaging measurements in a candle-like ignition setup (e.g., LOI) under atmospheric conditions using a 50 W propane testing flame. (**A**) Maximum temperature plot over time for a material selection; (**B**) Thermal imaging plot after 120 s ignition.

Measurements revealed a significant dependency of thermal conductivity properties and maximum temperatures observed in a candle-like ignition setup (e.g., LOI). Temperatures in the ignition zone increased quickly for PA6 formulations containing 25 wt.% EG or G, reaching a plateau-like characteristic after about one minute between 300 °C and 400 °C (Figure 9A,B). Since only little residue formation occurred, both EG and no-EG formulations showed comparable temperature developments over time. The slightly higher average temperatures as well as higher standard deviations measured for PA6/25 wt.% EG formulations can be partially attributed to graphite expansion, marking temperature peaks through glowing while protruding into the flame. Higher filling degrees of 50 wt.% and 70 wt.% G as well as 20/25 wt.% and 20/70 wt.% EG/G showed a substantially lower temperature development. Maximum temperatures measured in the heat impact zone were around 200 °C for 50 wt.% G and 20/25 wt.% EG/G as well as around 110 °C for 70 wt.% G and 20/50 wt.% EG/G. Thus, for the latter, pyrolysis temperatures were not reached within the given setup.

**Figure 10:**
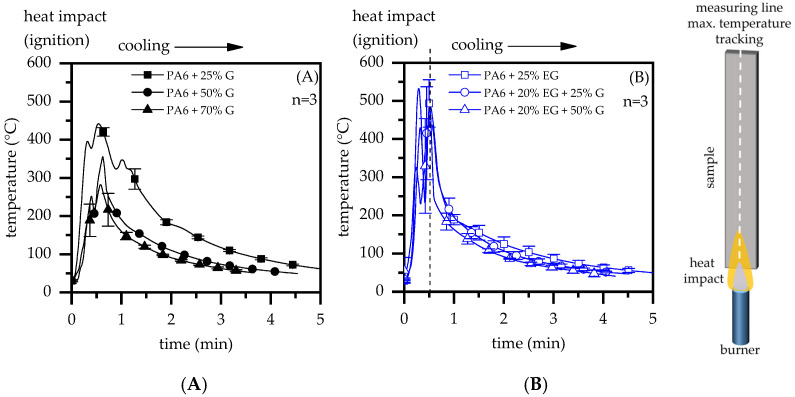
Thermal imaging measurements in a bottom-exposed ignition setup (e.g., UL-94) under atmospheric conditions using a 50 W methane testing flame. (**A**) Maximum temperature plot over time for a material selection; (**B**) Thermal imaging plot after 30 s ignition.

An entirely different behavior was found in a UL-94-like testing setup. UL-94 burning tests provide a substantially more severe heat impact scenario than candle-like ignition scenarios. By positioning a 50 W testing flame underneath a specimen, the heat impact occurs both at the flame-covered impact zone as well as by convective heating through hot upstreaming gases. This promotes quicker heat absorption and is also evident in temperature measurements conducted within this study (Figure 10). In a test setup similar to UL-94 (V-test), specimens were positioned vertically above a 50 W test flame, exposed two times each for 15 s and subsequently removed. In cases where ignition occurred, short blasts of compressed air were applied to extinguish the flame.

**Figure 11:**
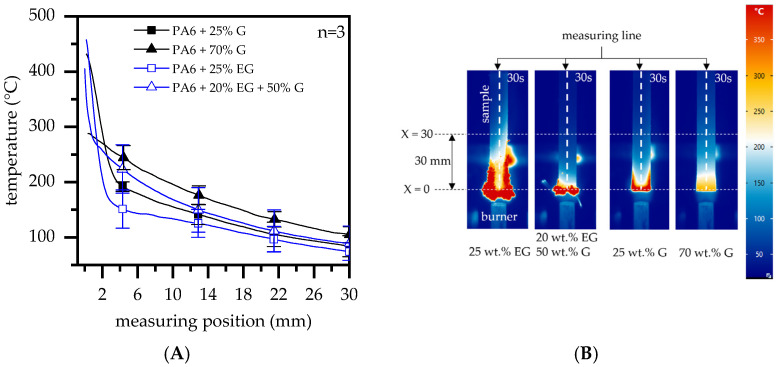
(**A**) Temperature distribution of selected samples at 30 s flame exposure measured midway along the length. All curves were averaged over three measurements; (**B**) thermal images and illustrated measuring position.

Compared to a previously discussed candle-like test setup, measured temperatures increased substantially more quickly. While PA6 formulations containing 25 wt.% G showed maximum temperatures in a range of 350 °C to 450 °C, heat accumulation in the ignition zone visibly decreased through improved thermal conductivity, evident in formulations containing filling degrees of 50 wt.% and 70 wt.% G. This fits well with findings discussed for the candle-like test setup above. PA6/EG and PA6/EG/G formulations revealed a considerable residue build-up with strong expansion, specifically in near-flame and edge regions (Figure 11B). High surface-to-volume ratios were the result of EG expansion, rapidly absorbing heat and thus appearing as high peak temperatures in thermal imaging measurements. While residual formation proceeded, larger part-areas were shielded from the flame. For higher filling degrees, improved thermal conductivity additionally provided strong heat dissipation capabilities that resulted in lower temperatures measured above shielded areas.

This is particularly evident when comparing thermal images taken after 30 s flame exposure. Figure 11A shows the vertical temperature distribution as illustrated in Figure 11B. Measurements were taken centrally between X = 0 mm, marking the lowest sample position of the original geometry, and X = 30 mm, above the lowest sample position. When comparing temperature profiles within the selected range, two fundamental characteristics could be identified:

(1) Characteristics regarding temperature distributions did not differ fundamentally for comparable filling degrees in PA6/G or PA6/EG formulations, but occurred at substantially different temperature levels. Since thermal conductivity properties of both formulations are comparable, we attribute this effect exclusively to an insulating residual effect. This provides thermal isolation between the flame and the heat input zone, as well as shielding for sample geometries above the voluminous residue, which would otherwise be convectively heated by a hot, uprising gas stream.
(2) Characteristics regarding temperature distributions differed fundamentally for different filling degrees, with higher filling degrees in particular showing fewer heat spots and a more homogeneous temperature distribution. This can be attributed to improved thermal conductivity properties (see also Figure 3), allowing heat to dissipate along the sample geometry while promoting convective cooling.

## 4. Discussion

Thermally conductive polymeric materials can be used for structural components with specific requirements in heat dissipation. Compared to metals, polymeric materials offer advantages in terms of economical processing, product weight or chemical resistivity, at the cost of a lower thermal conductivity and disadvantageous flame-retarding properties. To achieve satisfactory thermal conductivity, high filling ratios of thermally conductive particles are typically required. These occupy a majority of the potentially incorporable volume and thus hinder additional modifications with further functional additives. This study therefore investigated the use of expandable graphite as a multifunctional filler for thermally conductive polymeric materials, providing both flame retardancy and thermal conductivity properties. Systematically varied formulations based on graphite and expandable graphite incorporated in a PA6 matrix were investigated. Flame inhibitation effects due to heat conductivity properties and voluminous residue formation were evaluated separately.

Naturally, graphite-filled polymeric materials provide lower heat generation in cone calorimeter tests. Even though an improvement in residue formation was evident particularly for higher G-filler fractions, THE, MAHRE and pHRR decreased more or less directly proportionally to the substituted polymer fraction. An observed postponement of the ignition time can be attributed to dense residue formation, evaporating pyrolysis gases only locally when cracks occurred. In turn, pyrolysis gas streams partially bypassed piloted ignition, randomly postponing the ignition time. Formulations containing EG as a flame-retarding additive provided a disproportionally improved flame inhibitation mechanism by voluminous char residue formation. Important key figures characterizing the potential fire hazard improved substantially more than expected solely through fuel substitution effects, and are thus proven to be a superior flame inhibitation mechanism within the given test setup. It is thus concluded that G does not actively improve fire resistance in cone-calorimeter-like situations, but only provides lower fire loads by passive fuel substitution. Furthermore, since samples are thermally isolated in cone calorimeter tests and dissipated heat cannot be transferred into cooler areas, thermal conductivity properties were found to be negligible.

LOI and UL-94 test scenarios provide fundamentally different conditions. Only a small part of a specimen is exposed to a comparatively small test flame, allowing heat dissipation within the specimen geometry as well as convective heat exchange with the surrounding atmosphere. The ignition scenario is a decisive factor, technically controlling the potential heat impact. In candle-like ignition scenarios (LOI), a small flame is applied almost exclusively to the very top of a test specimen, thus limiting the potential heat impact to a small area. Convective heat evaporating as a hot upward gas stream does not interact with the test geometry and can thus be neglected. When exposing a test flame underneath a testing geometry (UL-94), the heat impact scenario becomes more severe. Larger surface areas are enclosed by an upward-directed flame, allowing a more intense heat exchange and thus quicker heating. Additionally, a hot gas stream leaving the visible flaming zone heats more remote specimen areas, reducing upstream cooling possibilities by atmospheric heat exchange.

Observations of heat development in a LOI-like test setup, as used within this study, showed decreasing heat accumulation intensities when higher G fractions were present. In particular, samples containing 50 wt.% and 70 wt.% G reached temperatures well below 250 °C, making ignition under atmospheric conditions improbable. PA6 containing only 25 wt.% G, exhibiting low thermal conductive properties, resulted in substantially higher peak temperatures around 350°C. Accordingly, the LOI values measured for 25 wt.%, 50 wt.% and 70 wt.% G were 27.8%, 40.2% and 76.9%, respectively. Furthermore, a good correlation between LOI values and thermal conductivity properties (thru-plane) was found. Specifically, filling degrees of up to 50 wt.% G exhibited a linear increase for LOI values and thermal conductivity. Similar correlations were found for formulations containing EG, yet on a generally higher level. Since applied fit functions run nearly parallel, the increase in LOI was attributed to higher thermal conductivity properties, whereas the distance between both functions was attributed to EG residue formation. Within the given setup, an LOI delta of about 10% was thus assessed to have been gained by thermal isolation effects provided through char residue formation.

UL-94 test setups provide a more severe heat impact scenario. Similar to scenarios found for LOI testing setups, higher thermal conductivity enabled dissipation to occur, showing accumulated heat to be transferred from the heat impact zone towards lower temperature areas. Consequently, PA6/G formulations with good thermal conductivity properties showed clear heat distribution in thermal images, implying lower temperature peaks during the flame exposure. Despite good thermal conductivity properties, only PA6/G formulations containing 70 wt.% G achieved a V0 classification. Moreover, when exposure times exceeded 2 × 10 s, samples tended to eventually burn without any signs of self-extinction. PA6 containing EG, on the other hand, provided an additional flame inhibitation effect. Strong expansion efficiently reduced the heat impact, such that samples containing 25 wt.%, as well as all tested formulations containing EG and G, achieved a V0 classification. Thus, thermal conductivity does not provide satisfactory flame-retarding properties for UL-94 like burning scenarios.

It was concluded that the thermal conductivity properties of polymeric systems can provide good flame inhibitation effects, although their efficiency strongly depends on the burning scenario. For ignition scenarios in which the ignition source is very small and heat can be effectively dissipated, it might be possible to avoid ignition. However, in fully developed fires that provide a high heat impact, good thermal conductivity properties alone are not capable of preventing ignition and do not provide sufficient self-extinguishing properties.

## 5. Conclusions

Expandable graphite was investigated as a multifunctional flame-retarding system for thermal conductive polymeric materials. Based on the experimental results, the following conclusions are drawn for this study:

- Expandable graphite showed excellent flame inhibitation properties for thermal conductive polymeric materials based on PA6 and graphite. A UL-94 V0 (1 mm) classification was achieved for filling degrees greater than or equal to 25 wt.%.
- Thermal conductivity did not influence burning characteristics measured in cone calorimeter tests. This was attributed to a heat trap setup where samples are thermally isolated, prohibiting heat exchange mechanisms.
- For LOI testing scenarios, evidence was found that thermal conductivity contributes greatly to the LOI value achieved. Up to a filling degree of 50 wt.% graphite, correlations between the LOI measured and thermal conductivity properties provided a linear characteristic. Higher filling degrees did not fit the linear characteristic, most probably due to oxidizing graphite.
- In UL-94 testing setups, good thermal conductivity properties improved heat dissipation measured at the sample surface for PA6/G formulations. However, since the heat impact scenario is more severe than in candle-like setups, its effect on flame inhibitation probabilities is more limited. Only high weight loads of 70 wt.% graphite achieved V0 classification, but eventually burned when exposure times exceeded 2 × 10 s, without self-extinguishing characteristics.
- We conclude that thermal conductivity can affect inflammation processes, but its efficiency depends strongly on the ignition scenario. The expansion of expandable graphite provides excellent flame retardancy effects in thermal conductive polymeric materials, since no additional additive is needed to gain both properties. Thus, for the development of thermally conductive polymeric materials that need to provide high flame retardancy classifications, a combination of graphite and expandable graphite might be a solution worth considering.

## Figures and Tables

**Table 1:** Materials properties—summary.

Material	Trade Mark	Properties
polyamide 6 (PA6)	B27E (BASF SE)	melting temperature 230 °C; density 1.13 g/cm³, MVR 130 270 °C/5 kg
expandable graphite (EG)	GHL PX 95 HT 270 (LUH GmbH)	70% > mesh 50; temperature stability 270 °C; density 2.17 g/cm³; purity >95% C; expansion volume min. 200 g/mL
graphite (G)	GHL 3394 (LUH GmbH)	70% > mesh 50; density 2.17 g/cm³; purity >95% C

**Table 2:** TGA measurement—summary.

	PA6 wt.%	EG wt.%	G wt.%	EG(G) vol.%	T_m_ Peak °C	T_99%_ Onset °C	DTG-Peak °C	Residue %
a	100			0	220 ± 3	385 ± 3	472 ± 2.3	0.9 ± 0.1
	-	100			-	307 ± 3	349 ± 1.2	83 ± 1.1
b2	85	15		8	219 ± 1	332 ± 2	465.2 ± 1.1	14.2 ± 1.8
b4	75	25		15	221 ± 1	316 ± 2	457.9 ± 2.7	23.6 ± 1.2
c2	85		15	8	221 ± 1	385 ± 2	471.0 ± 0.3	14.9 ± 0.3
c4	70		25	15	221 ± 2	386 ± 3	469.2 ± 0.7	22.6 ± 0.3
d1	55	20	25	30	220 ± 1	329 ± 3	455.7 ± 1.7	42.8 ± 0.3
d2	30	20	50	56	221 ± 1	328 ± 1	449.2 ± 0.6	67.6 ± 0.1
e1	50		50	35	220 ± 3	390 ± 2	469.5 ± 0.9	50.4 ± 0.4
e2	30		70	56	220 ± 2	392 ± 3	464.4 ± 0.9	69.5 ± 0.6

**Table 3:** Thermal conductivity measurements—summary.

	PA6 wt.%	EG wt.%	G wt.%	EG(G) vol.%	Thermal Conductivity (x) W/mK	Thermal Conductivity (z) W/mK
a	100			0	0.27 ± 0.01	
b2	85	15		8	0.56 ± 0.04	
b4	75	25		15	0.89 ± 0.05	
c2	85		15	8	0.61 ± 0.05	
c4	70		25	15	0.90 ± 0.05	
d1	55	20	25	30	1.79 ± 0.30	
d2	30	20	50	56	7.50 ± 0.22	31.54 ± 0.81
e1	50		50	35	2.08 ± 0.16	
e2	30		70	56	9.15 ± 1.20	30.97 ± 0.71

**Table 4:** Summary—fire testing results Cone calorimeter; 50 kW/m².

	PA6 wt.%	EG wt.%	G wt.%	EG/G vol.%	Mass g	t_ign_ s	pHRR kW/m²	THE MJ/m²	MAHRE kW/m²	TSP m²
a	100			0	46 ± 0.2	83 ± 3	706 ± 48	125 ± 1	213 ± 23	8.9 ± 0.9
b1	90	10		6	47 ± 0.2	54 ± 2	359 ± 23	103 ± 2	187 ± 3	7.4 ± 0.1
b2	85	15		8	48 ± 0.1	56 ± 3	166 ± 30	26 ± 3	48 ± 1	2.3 ± 0.2
b3	80	20		12	50 ± 0.3	58 ± 3	122 ± 3	24 ± 2	44 ± 4	1.6 ± 0.2
c1	90		10	6	47 ± 0.2	62 ± 3	406 ± 27	111 ± 2	210 ± 4	6.6 ± 0.4
c2	85		15	8	48 ± 0.3	64 ± 2	474 ± 8	112 ± 1	211 ± 4	7.6 ± 0.5
c3	80		20	12	50 ± 0.5	89 ± 5	441 ± 50	105 ± 2	188 ± 13	7.3 ± 1.1
d1	55	20	25	30	55 ± 0.5	91 ± 4	50 ± 14	7 ± 6	176 ± 8	6.8 ± 1.1
d2	30	20	50	56	66 ± 0.5	0 ± 0	0 ± 0	0 ± 0	0 ± 0	0 ± 0
e1	50		50	35	59 ± 0.4	180 ± 11	112 ± 56	66 ± 3	129 ± 8	4.8 ± 0.9
e2	30		70	61	67 ± 0.5	191 ± 10	193 ± 56	27 ± 5	57 ± 12	2.2 ± 0.9

**Table 5:** UL-94 and LOI test results for 1, 2 and 4 mm thick samples.

	PA6 wt.%	EG wt.%	G wt.%	EG+G vol.%	UL-94 1 mm	UL-94 2 mm	UL-94 4 mm	LOI 1 mm %	LOI 2 mm %	LOI 4 mm %
a	100			0	HB	HB	V2	24.2 ± 0.1	24.5 ± 0.2	26.1 ± 0.2
b1	90	10		6	HB	HB	-	31.3 ± 0.2	26.7 ± 0.2	
b2	85	15		8	HB	HB	HB	34.3 ± 0.1	33.4 ± 0.2	35.9 ± 0.2
b3	80	20		12	V2	V2	-	36.9 ± 0.1	34.4 ± 0.3	
b4	75	25		15	V0	V0	V0	41.8 ± 0.2	39.1 ± 0.2	42.5 ± 0.2
c1	90		10	6	HB	V2	-	21.2 ± 0.2	22.8 ± 0.2	
c2	85		15	8	HB	HB	HB	22.4 ± 0.3	24 ± 0.2	24.7 ± 0.2
c3	80		20	12	HB	HB	-	22.8 ± 0.2	24.4 ± 0.2	
c4	75		25	15	HB	HB	HB	24.6 ± 0.2	25.2 ± 0.2	27.8 ± 0.1
d1	55	20	25	30	-	-	V0	-	-	50.5 ± 0.2
d2	30	20	50	56	-	-	V0	-	-	>80
e1	50		50	35	-	-	HB	-	-	40.2 ± 0.2
e2	30		70	61	-	-	V0	-	-	76.9 ± 0.2
